# Evaluation of *Bacillus velezensis* for Biological Control of *Rhizoctonia solani* in Bean by Alginate/Gelatin Encapsulation Supplemented with Nanoparticles

**DOI:** 10.4014/jmb.2105.05001

**Published:** 2021-08-12

**Authors:** Mojde Moradi-Pour, Roohallah Saberi-Riseh, Keyvan Esmaeilzadeh-Salestani, Reza Mohammadinejad, Evelin Loit

**Affiliations:** 1 Department of Plant Protection, Faculty of Agriculture, Vali-e-Asr University of Rafsanjan, Rafsanjan 7718897111, Iran; 2 Chair of Crop Science and Plant Biology, Institute of Agriculture and Environmental Sciences, Estonian University of Life Sciences, Fr. R. Kreutzwaldi 1, EE51014 Tartu, Estonia; 3 Research Center of Tropical and Infectious Diseases, Kerman University of Medical Sciences, Kerman 7618866749, Iran

**Keywords:** Alginate/Gelatin, biological control, encapsulation, carbon nano tube, microbiology, PGPR

## Abstract

Plant growth promoting rhizobacteria (PGPR) are a group of bacteria that can increase plant growth; but due to unfavorable environmental conditions, PGPR are biologically unstable and their survival rates in soil are limited. Therefore, the suitable application of PGPR as a plant growth stimulation is one of the significant challenges in agriculture. This study presents an intelligent formulation based on *Bacillus velezensis* VRU1 encapsulation enriched with nanoparticles that was able to control *Rhizoctonia solani* on the bean. The spherical structure of the capsule was observed based on the Scanning Electron Microscope image. Results indicated that with increasing gelatin concentration, the swelling ratio and moisture content were increased; and since the highest encapsulation efficiency and bacterial release were observed at a gelatin concentration of 1.5%, this concentration was considered in mixture with alginate for encapsulation. The application of this formulation which is based on encapsulation and nanotechnology appears to be a promising technique to deliver PGPR in soil and is more effective for plants.

## Introduction

Human life is entirely dependent on plants; to the extent that 90% of the nutrients needed by humans are supplied by plants. Therefore, any factor disrupting the production of agricultural products will directly affect human nutritional needs. Pathogenic microorganisms, including fungi, are considered the most critical pathogens and cause direct damage and disease in plants.

Bean is an essential nutritional plant and a rich source of protein. One of the main causes of bean root and crown rot around the world is Rhizoctonia which causes many damages to bean fields every year. As the destructive effects of chemical pesticides became apparent and public protests over the use of these substances increased, tendency towards other methods such as biological control methods rose [1].

Biological control of plant diseases is one of the most essential practices in sustainable agricultural production systems [2, 3]. With growing concern about introducing biocontrol agents into the rhizosphere, it becomes of particular importance to characterize effective biocontrol agents under various conditions. Biocontrol agents are frequently ineffective due to microbial competition or adverse environmental conditions [4]. The use of antagonistic bacteria under field conditions may have little or no effect on plant disease control. Prior to releasing biological control agents, measures are needed to ensure the stability, efficiency, and growth of biocontrol agents in laboratory and natural conditions (greenhouse and field) against plant diseases. Hence, the suspension of antagonist bacteria must be stabilized in individual carriers and the formulation. The advantages of the formulation include ease of use, ease of transportation, long-term storage, increasing farm efficiency, and commercialization [5, 6].

Agriculture sector has always been at the top obtaining end of technology advancement in recent years. The application of a gradual release system based on encapsulation technology is considered a good process for storing and delivering beneficial bacteria [7]. To increase the efficiency and survival of biocontrol agents, scientists have proposed different methods of encapsulation. Encapsulation of bacteria creates a wall-like layer which controls the release of microorganisms, protects them, and guarantees their functional ability [8]. In the past decades, biopolymers have been used as wall materials for encapsulation in different industries especially agriculture [9]. Natural polymers are produced and extracted by biological agents such as microorganisms, fauna, and flora. The result of Gagne-Bourque *et al*. [10] indicated that *Bacillus subtilis* B26 encapsulated in alginate and pea protein as wall materials. The provision of healthy and nutritious food and agricultural products to continue living has always been one of the challenges of human societies. The adoption of technologies with the purpose of increasing the quantity and quality of agricultural products can be an excellent strategy to address these challenges. One of these technologies is nanotechnology. The use of silicon nanoparticles enhances the net photosynthetic rate, stomatal conductance, intercellular CO_2_ decreased in transpiration rate, water use efficiency, and content yield [11]. One of the most important effects of carbon nanotubes on plant seeds is increasing the percentage and speed of germination [12]. Carbon nanotubes facilitate the entry of oxygen into the seeds by penetrating them. It is also possible that carbon nanotubes can help water enter the cells by affecting the water channels in cell membrane and regulating their action [13]. The results of our previous research showed that *Bacillus velesensis* plays an effective role in increasing soil quality by secreting secondary metabolites and thus enhancing plant growth and boosting its resistance to plant pathogens [14]. This study aimed to obtain an intelligent formulation of *B. velesensis* bacteria that can increase the survival and efficiency of biocontrol agents in soil conditions with gradual release and the synergistic effect of silica nanoparticles and carbon nanotubes that have a significant influence on controlling *Rhizoctonia solani* on the bean.

## Experimental

### Materials

*B. velezensis* VRU1 were obtained from the biological control collections of the Vali-e-Asr University of Rafsanjan, an isolate of *R. solani* was selected from Khorasan Razavi Agricultural and Natural Resources Research. Carbon nanotubes (CNT) was obtained from Iran Nanotechnology Innovation Council and synthesis of SiO_2_ nanoparticles was performed in the nanotechnology laboratory of the Vali-e-Asr University of Rafsanjan (Iran). potato dextrose agar (PDA), nutrient agar (NA), L-tryptophan, indole-3-acetic acid (IAA), CAS-agar, and other materials used in this research were purchased from Merck company (Germany).

### Methods

#### Zone of Inhibition Test

This test was performed using the petri dish in vitro according to the method of Keel *et al*. [15]. *B. velezensis* VRU1 was cultured at half a centimeter from the edge of the petri dish containing PDA medium. An hour later, mycelia plug of *R. solani* was placed in the center of the petri dish. Sterile distilled water was used as a control. Petri was completely covered with paraffin. The plates were incubated at 25°C for five days. A comparison of the inhibitory zone diameter with the control indicates the degree of bacterium biocontrol against the pathogenic fungus.

#### Determination of Indole Acetic Acid (IAA) Production

The method of Patten and Glick [16] was used to do an assay of IAA. Bacterial strain culture was inoculated in nutrient broth medium for 48 h, and then 50 ml of it was added to nutrient broth (NB) medium containing 200 mg/l L-tryptophan and incubated at 28 ± 2°C for 72 h. The cell cultures were centrifuged at 3,000 rpm for 10 min and 2 ml of the supernatant phase was mixed with 4 ml of Solawaski's reagent (50 ml of 35% perchloric acid and 1 ml of 0.5 M FeCl_3_). Observation of pink color demonstrates IAA production. Samples were kept in a dark place for 20 min and optical density was measured at 535 nm using a spectrophotometer.

#### The Siderophore Production Assay

The ability of *B. velezensis* VRU1 to produce iron-binding compounds of siderophore-type was assayed on petri dishes containing CAS-agar medium according to the method of Arora and Verma [17]. Bacterial cells were cultured on CAS-agar plates using a sterile loop and incubated at 28°C for five days. Observation of orange zones around the colony was considered as siderophore–production.

#### Mineral Phosphate Solubilization by Bacteria

The ideal culture medium for mineral phosphate solubilization was obtained from Son *et al*. [18]. To determine phosphate solubilization, Pikovaskya medium (glucose, 10 g/l; ammonium sulphate, 0.5 g/l; sodium chloride, 0.2 g/l, potassium chloride 0.2 g/l, manganous sulphate, 0.002 g/l, magnesium sulphate, 0.1 g/l; tri-calcium phosphate, 5 g/l, and ferum sulphate, 0.002 g/l) agar plates were inoculated with the bacterial cell. After five days of incubation at 28°C, appearance of a clear zone around bacterial culture indicated phosphate solubilization capacity.

#### Protease Activity

Protease activity was done according to the method of Berg *et al*. [19] with modifications. Bacterial cells cultured in skim milk agar (50 ml sterilized skim milk mixed with 100 ml NA and 4% agar at 55°C) and incubated at 28°C. After two days, the clearing zone around the bacterial culture indicated the production of protease enzyme.

#### Chitinase Production

The chitinase activity of *B. velezensis* VRU1 was assayed by a modified method of Toharisman *et al*. [20] To prepare chitinase culture medium, 5 g of colloidal chitin and 18 g of agar to M9 medium (0.65 g Na_2_HPO_4_, 1.5 g KH_2_PO_4_, 0.25 g NaCl, 0.5 g NH4Cl, 0.12 g MgSO_4_·7H_2_O 0.005 g of CaCl_2_) were added to one liter of distilled water and after autoclaving the bacteria were cultured on it. It was incubated for ten days at 28°C. The appearance of a colorless halo around the bacterial colony indicates the bacterial strains produce chitinase.

### Preparation of Nanocomposite Beads and Study of Their Properties

#### Preparation of Culture Medium

The *B. velezensis* VRU1 was cultured in liquid culture medium (0.5 g of beef extract, 1 g of peptone, 0.5 g of sodium chloride per liter) and incubated for 72 h at 28°C on a rotary shaker at 130 rpm.

#### Investigation of the Effect of Nanoparticles on Bacterial Growth

Antimicrobial activity of the nanoparticles used in encapsulation was determined by using the agar well diffusion assay [21]. Four wells (5 mm) were created in four regions on plates containing NA medium with a cork borer. *B. velezensis* VRU1 was spread onto plates and different concentrations of SiO_2_ nanoparticles and CNT were added in the well. Sterile water was used to control the treatment. After two days of incubation, the inhibition zone around the wells showed the antimicrobial activity of nanoparticles.

#### Preparation of *B. velezensis* VRU1 Nanocomposite Beads

The method presented by Tu *et al*. [22] was used to prepare the beads. To summarize, populations of bacterial suspension (10^10^ CFU/ml) were added to 80 ml of 2% sodium alginate and various percentages of gelatin (0, 0.5, 1, 1.5, 2, 2.5%). Then, encapsulation process was started by adding 20 mM SiO_2_ nanoparticle, 40 μg/ml carbon nanotubes, and 2% CaCo_3_ nanoparticles. After adding soybean oil and Span 80, it was stirred for 15 min. When the solution became uniform, 500 μl of acetic acid was added to it and it was placed on the steering for another 15 min. Finally, by adding CaCl_2_, the encapsulation process was completely performed. After centrifugation, the nanocomposite beads were washed with sterile physiological saline and stored at 4°C.

#### Microscopic Examination of the Beads Structure

To evaluate the capsule structure, imaging was performed by using a scanning electron microscope (SEM) (EM 320) after the beads dried at 45°C.

#### Determination of Moisture Content in Nanocomposite Beads

This test was performed based on the method of Tu *et al*. [22] with some modifications. One gram of wet nanocomposite beads was carefully weighed (W_w_) and dried in an oven at 40°C, then the dry weight of the granules was measured (W_d_) and their moisture content was calculated by the following formula:

Moisture content= (W_w_ – W_d_) / W_w_ ×100

#### Swelling Percentage of the Beads

One gram of dry nanocomposite beads (W_d_) was weighed and added to 10 ml of sterile physiological saline. After 24 h, excess water was removed using filter paper. The weight of the nanocomposite beads (W_w_) was accurately measured by a sensitive scale and the swelling rate was calculated based on the following formula:

Moisture content= (W_w_ – W_d_) / W_w_ ×100

#### Evaluation of Encapsulation Efficiency

To evaluate the number of bacteria encapsulated in the beads, one gram of the prepared nanocomposite beads was added to 10 ml of physiological serum. After one hour, it was cultured on NA medium and placed in 28°C incubator for 24 h [23]. Colony count was performed on NA medium and encapsulation efficiency was calculated by the following formula:

Encapsulation efficiency: Bacteria in the capsules (CFU/g) / Bacteria added to formulation (CFU/ml) × 100

#### Measurement of Release and Viability of Bacteria in Soil

This experiment was performed based on the modified method of Wu *et al*. [23]. For this purpose, the bacteria became resistant to the antibiotic rifampicin (75 μg/ml) [24], and nanocomposite beads with different concentrations of gelatin were buried in sterile soil. The soil was placed at room temperature for 60 days. To maintain soil moisture and bacterial release from the beads, sterile distilled water was sprayed as necessary. At different time intervals, one gram of soil was weighed and added to 10 ml of sterile distilled water. After preparing the dilution series, culture was performed on NA medium containing rifampicin antibiotic and the colonies were counted.

#### Greenhouse Experiments

Bean seeds were surface sterilized in 0.5% sodium hypochlorite for three minutes, washed with distilled water, and placed on agar medium for 48 h. The treatments were applied to sterilized soil in pots in 4 replications (see table 1) and five germinating seeds were placed in each pot. The pots were kept in a greenhouse at a temperature of 25-29°C and to inoculate with *R. solani*, wheat colonized with *R. solani* was added. To add the bacterial suspension, a suspension of 24-h culture of bacteria with a concentration of 10^10^ CFU/ml in sterile distilled water was prepared and 10 ml/kg of potting soil was added. Pots containing nanoformulation treatments were inoculated with ten grams of nanocomposite beads of bacterial strain per kilogram of soil. After 60 days, the disease symptoms were evaluated. The severity of *R. solani* pathogenicity was recorded using the scoring described by Nelson *et al*. [25].

0= healthy

1= 1-10% infection of hypocotyls

2= 11-30% infection of hypocotyls

3= 31-50% infection of hypocotyls

4= 51-80% infection of hypocotyls $\text{\%DI=}\frac{\sum_{\text{i-1}}^{\text{n=1}}{\text{N}}_{\text{t}}\text{×S}}{{\text{N}}_{\text{o}}\text{×5}}\text{×100}$

5= plant dead.

S: Disease scale between 0-5

No: Total number of bean plants

Nt: number of bean plants in treatment

Control of disease in treatment%= Disease severity in treatment% - Disease index in infected control%

**Statistical Analysis**. The data of bacterial release, moisture content, swelling ratio, and encapsulation efficiency were analyzed in one-way ANOVA. Significantly, SAS 9.1 (SAS Institute, Inc, USA) was used for data analysis and comparing means. Each test was performed with three replications.

## Results and Discussion

### Zone of Inhibition Test

**Fig. 1:**
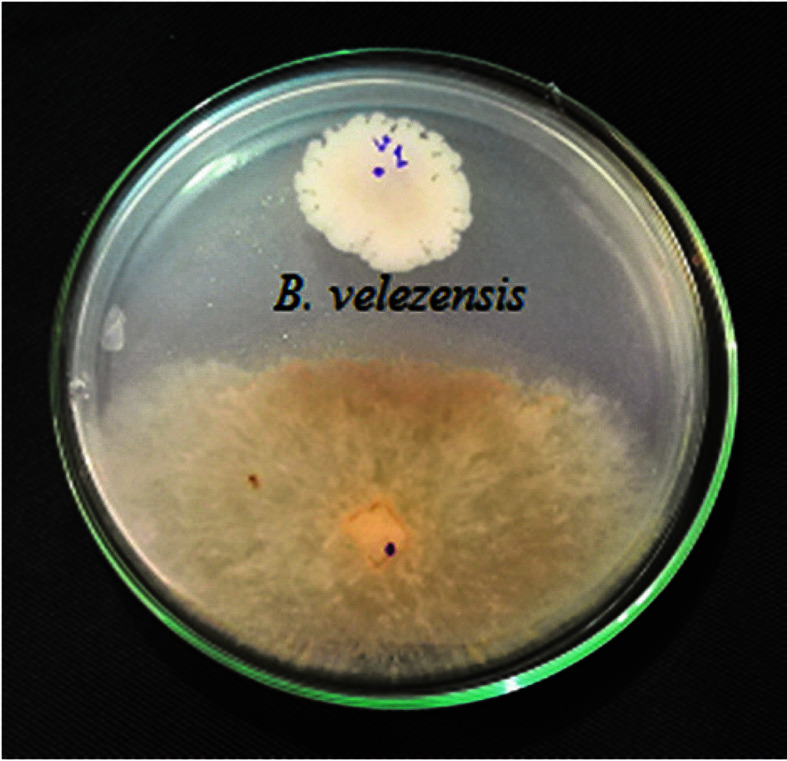
Inhibition zone of *B. velezensis* VRU1 against *R. solani*.

The results indicated that *B. velezensis* VRU1 has a significant inhibition zone of the *R. solani* mycelia growth, representing antifungal metabolites secreted by this strain (Fig. 1).

### Proteases and Chitinase Production

**Fig. 2:**
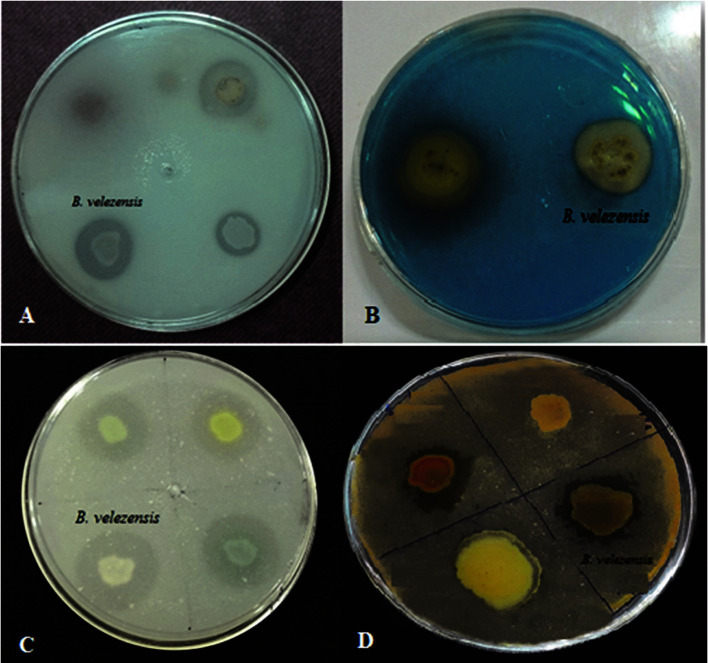
A: Solubilize inorganic phosphate ability; B: Siderophore production; C: Proteases Production; D: Proteases and Chitinase Production.

This strain was also able to produce proteases and chitinase. Appearance of a colorless halo around the bacterial colony indicates the production of these enzymes (Fig. 2). McQuilken and Gemmell reported that proteases and chitinase might play an important role in penetrating and lysing the cell walls of *R. solani* [26]. Since the main components of the *R. solani* cell-wall are composed of chitin [27], the chitinase enzyme produced by *B. velezensis* VRU1 can be effective in the lysis of this fungus cell-wall.

### IAA Production

The quantitative analysis of IAA was performed using nutrient broth medium with L- tryptophan (200 mg/l). Results indicated that *B. velezensis* VRU1 was able to produce 28.3 μg/ml IAA in this medium. The IAA phytohormone produced by plant growth promoting rhizobacteria (PGPR) is an effective molecule in plant interactions, phytostimulation processes, and pathogenesis [28]. Auxin is involved in the development, growth, and defense mechanisms of plants; it is also effective in root enhancement and this can lead to more nutrients provision for plants [29]. Bari *et al*. [30], claimed that the auxin might enhance plant growth through cell enlargement, cell division, root initiation, and increased growth rate.

### Solubilize Inorganic Phosphate Ability

The ability of *B. velezensis* VRU1 to solubilize inorganic phosphate was done on Pikovaskya agar plates qualitatively. Results showed that the investigated strain had the potency to solubilize in an inorganic phosphate efficiently (Fig. 2). Phosphate solubilizing microorganisms, such as bacteria, cause increasing P availability for plants by solubilization of insoluble soil phosphate into soluble forms available for plant growth; therefore, they act as biofertilizers. Shi *et al*. [31] revealed that the yield of different crops increased due to phosphate solubilizing bacteria.

### Siderophore Production

Siderophore is a small high-affinity iron (III)-chelator compound produced and secreted by microorganisms such as bacteria and fungi [32]. The siderophore production of *B. velezensis* VRU1 was investigated and after five days, an orange halo zone (1.6 cm in diameter) which indicates the studied bacterium’s ability to produce siderophore (Fig. 2), was observed around the bacterial colony. Plants can absorb Fe^2+^ from bacterial siderophores via various mechanisms [33].

### Nanocomposite Beads

**Fig. 3:**
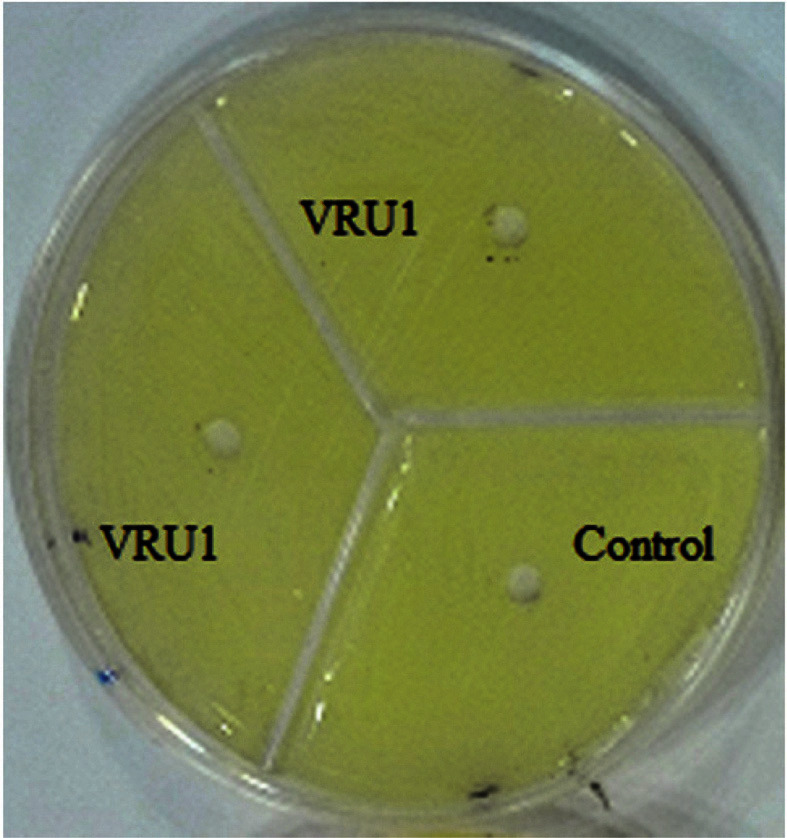
The effect of nanoparticles on bacterial growth.

**The effect of Nanoparticles on Bacterial Growth.** According to the results, an inhibition zone was not observed around the wells containing nanoparticles (Fig. 3); on this account, it can be claimed that the nanoparticles used (CNT and SiO_2_) in this study had no harmful effects on *B. velezensis*. Therefore, with the important role of these nanoparticles in agriculture, it is expected that they can have synergistic effects with probiotic agents in controlling plant diseases. Moradi *et al*. [34] reported alginate/gelatin nanocomposite beads of *Pseudomonas fluorescens* VUPF5 and T17-4 have significant effects on controlling potato dry rot.

**Fig. 4:**
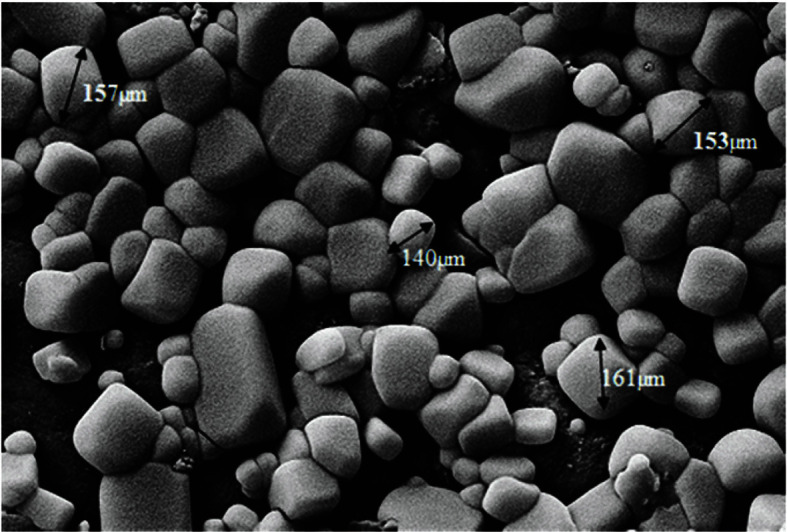
SEM image of the alginate–gelatin nanocomposite beads.

**SEM analysis of the Alginate–Gelatin Nanocomposite Beads**. The surface morphologies of the nanocomposite beads utilizing emulsification method were shown in Fig. 4. In Fig. 4 shown in the emulsification method of nanocomposite beads were found to exhibit a spherical appearance and they have an average diameter of about 150 μm.

### Determination of Moisture Content and Swelling Ratio in Nanocomposite Beads

**Fig. 5:**
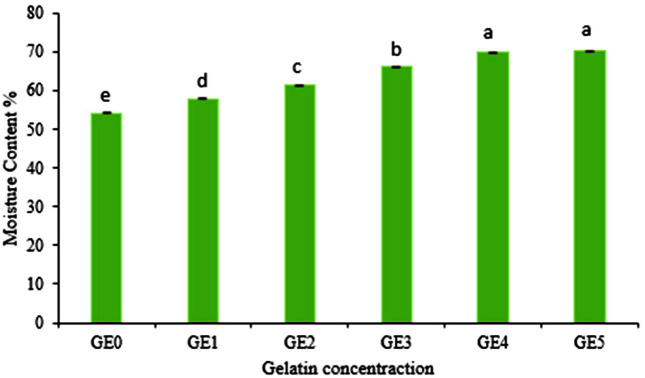
Moisture content of *B. velezensis* VRU1 nanocomposite beads prepared by various concentration of gelatin.

**Fig. 6:**
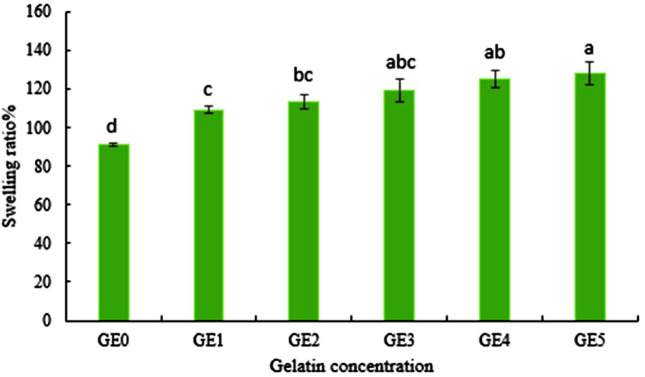
Swelling ratio of *B. velezensis* VRU1 nanocomposite beads prepared by various concentration of gelatin.

ANOVA results of moisture content indicated that this parameter was significantly affected by the concentration of gelatin (*p* < 0.01). The results showed that the moisture content of nanocomposite beads of *B. velezensis* VRU1 was variable from 53.93% to 70.14% with different amounts of gelatin (Fig. 5). Tu *et al*. [22] reported that the moisture content of microcapsules increased by increasing the percentage of gelatin. According to the results, swelling ratio of *B. velezensis* VRU1 nanocomposite beads varied from 91.1 to 128.16. It was observed that by increasing the concentration of gelatin, the moisture content of the nanocomposite beads increased (Fig. 6). This is perhaps because gelatin is water soluble and since it absorbs more water, it improves the moisture content of the beads [34]. The results indicated that as the gelatin concentration increases, the swelling ratio of beads rises accordingly. It can be due to the increasing number of intra-molecular connections of alginate and gelatin, or because of the number of interactions between the O=H groups of gelatin and water.

### Evaluation of Encapsulation Efficiency

**Fig. 7:**
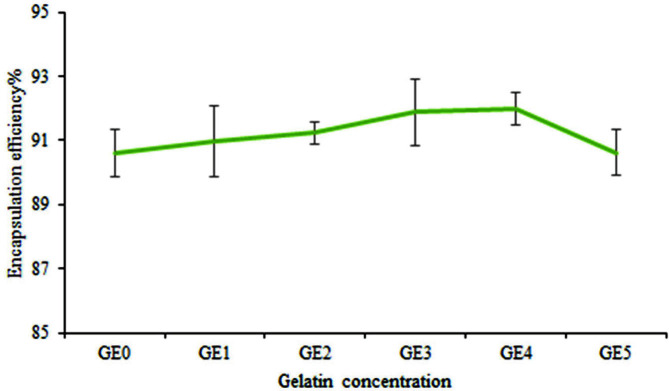
Encapsulation efficiency of *B. velezensis* VRU1 nanocomposite beads prepared by various concentration of gelatin.

The results showed that different concentrations of gelatin affected encapsulation efficiency. The efficiency of encapsulation of alginate mixture with varying concentrations of gelatin is presented in Fig. 7. The maximum encapsulation efficiency in bacterial strain beads was observed in an alginate mixture with 1.5% gelatin. The increase of gelatin amount, initially enhanced the efficiency of encapsulation; however, it then reduced. Tu *et al*.[22] showed that the concentration of 1.5% gelatin in combination with alginate has the highest encapsulation efficiency.

### Measurement of Release and Viability of Bacteria in the Soil

**Fig. 8:**
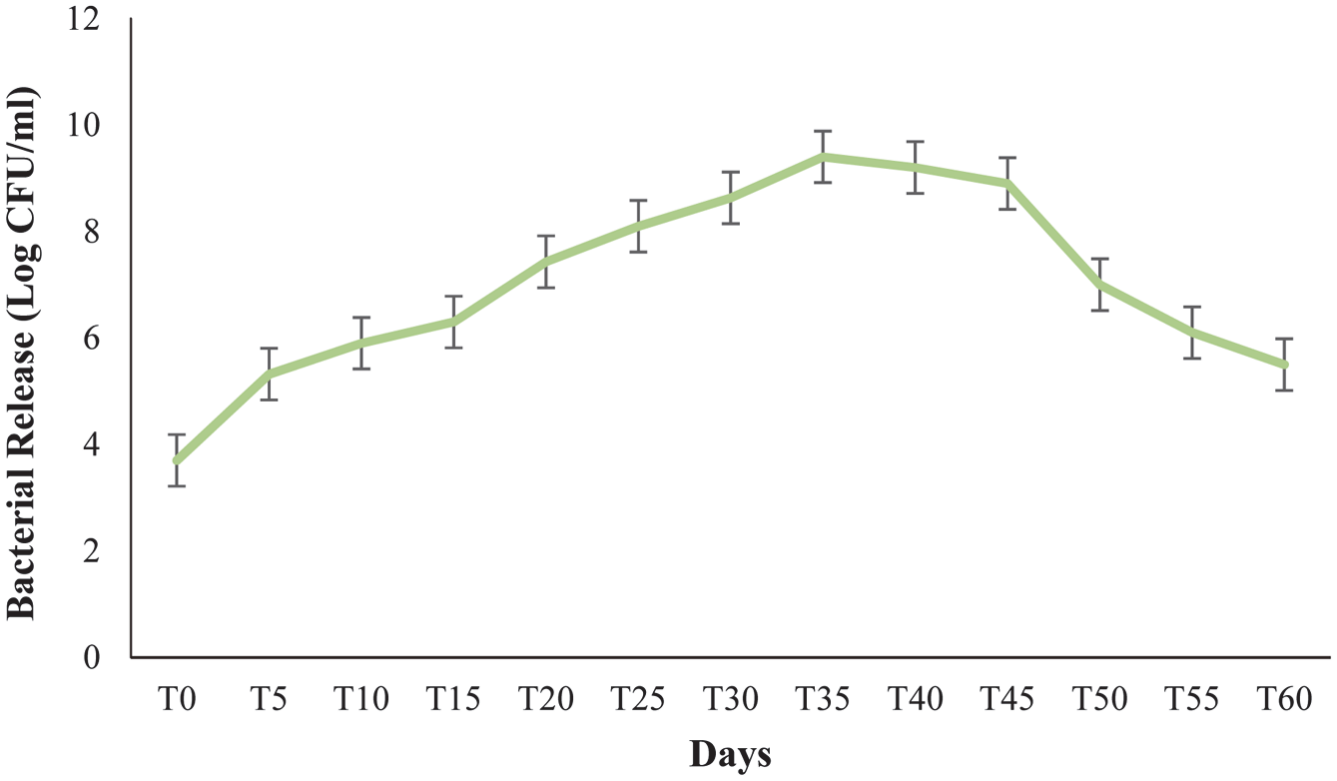
Effect of various gelatin concentrations in nanocomposite beads on viability and release of *B. velezensis* VRU1 in soil.

Fig. 8 shows the effect of various concentrations of gelatin (0-2.5%) used in the formulation, on the release and survival of *B. velezensis* VRU1 at room temperature for 60 days. The results showed that the bacteria population released from beads and their interaction were significantly affected by various concentrations of gelatin. The bacterium initially became resistant to the antibiotic Rifampicin. The number of released bacteria was determined by counting the colony on the nutrient agar medium containing antibiotics. After the preparation of the formulation, the number of *B. velezensis* VRU1 released from nanocomposite beads increased quickly until the 35^th^ day; the highest bacterial release was recorded on this day and after the 35^th^ day, it started to decrease. This result is probably due to environmental conditions, and the beginning of increase in bacteria death rate. Results showed that the bacteria survival rate was affected by various concentrations of gelatin, and the degradation rate of nanocomposite beads matrix is directly related to the biological activity of soil organisms. Due to excellent hydrophilic properties of gelatin (it increases the pores in the capsule by water absorbing and is effective in bacteria release process), we used gelatin and alginate mixtures. The release of biological bacteria can be controlled by adding biodegradable supplements into the matrix. Wu *et al*. [23], reported that after adding biodegradable starch to the capsule matrix of *R. planticola* Rs-2, bacteria release from microcapsules was very rapid. Alginate beads containing 1.5% gelatin could release the biggest number of bacteria on the 35^th^ day; this is probably due to their high efficiency in encapsulation and bacteria protection. Thus, the suitable filler concentrations can assist in the bacteria release. Results of the present study are similar to the findings of Van Elsas *et al*. [39] which showed that *P. fluorescens* encapsulated in alginate beads, could improve bacteria effectiveness and colonization of wheat roots.

### Greenhouse Experiments

**Fig. 9:**
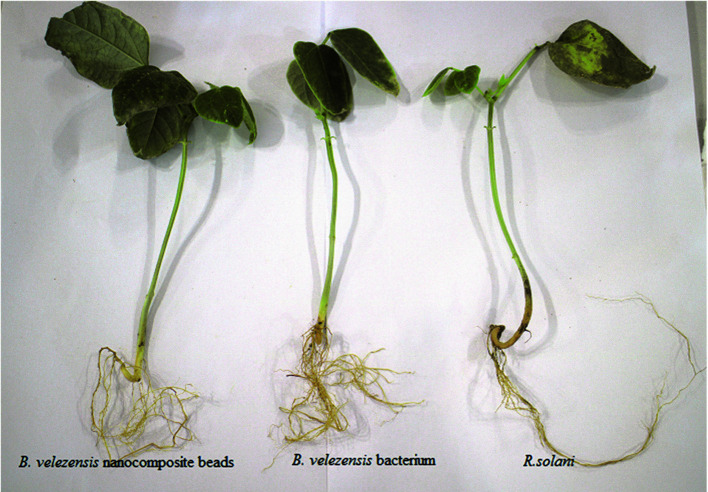
The effect of *B. velezensis* VRU1 and their nanoformulations on control of R.solani on bean plants.

After 60 days of treatment, it was observed that all the treatments related to this study significantly reduced the disease severity percentage compared to the control. Based on Duncan's mean comparison at 1% level, *B. velezensis* VRU1 nanocomposite beads treatment will be in group b with 96.33% disease control, and nanocomposite beads without bacteria treatment which showed the smallest effect with 25% disease severity reduction, belong to group d (Table 2). In plants inoculated with *B. velezensis* VRU1 nanocomposite beads, the percentage of disease control was 26% compared to free *B. velezensis* VRU1 (Fig. 9). Results of this study also revealed that nanocomposite beads caused an increase in plant growth factors (Table 3). The dry weight of shoots and roots in plants treated by nanoformulation were 1.84 g and 1.07 g respectively, while 0.4 g and 0.25 g of weight were recorded for the control.

## Conclusion

Plant growth-promoting rhizobacteria are beneficial microorganisms and produce many metabolites such as siderophores, chitinase, protease, cellulase, antibiotics, 1-aminocyclopropane-1-carboxylic acid (ACC) deaminase, etc [40]. Numerous PGPR formulations have been developed with various applications around the world [41]. Research based on new formulations which are compatible with environmental conditions should be a priority in sustainable agriculture [42]. Innovation in formulation and survival rate determination of bacteria are the main steps in developing bacterial inoculants [43]. This study presents a new formulation that increases the survival and efficiency of bacterial agents in unfavorable environmental conditions by gradual release. Also, the SiO_2_ nanoparticle used in the capsule wall induces resistance in the plant, creates a layer inside the plant cell wall, and controls the pathogens penetration into the host plant tissue. Nanotechnology is a new science that has attracted many researchers' attention [44]. SiO_2_ nanoparticles act as a carrier and can bind to chemical compounds to guide them into plant cells [45] and improve plant germination and growth [45]. SiO_2_ nanoparticles increased seedling growth, root diameter, root length, and lateral roots on Changbai larch [46]. Carbon nanotubes enhance seed germination, development, and growth of plants [47]. In numerous studies researchers have reported that carbon nano tubes have the ability to penetrate the seed and increase germination and plant growth [48-52]; they also bind to chemicals and facilitate their entry into the plant [53]. CNT induces water, Fe^3+^, and Ca^2+^ nutrients uptake efficiency in plants; this can lead to germination raise and plant development [54]. Therefore, it can be claimed that these nanoparticles probably bond to chemical compounds (*e.g.*, auxin) secreted by bacteria, and improve plant growth by increasing chemical uptake by the plant. This intelligent formulation is presented in a way that the bacteria trapped in it control the release of plant pathogens by gradual release; also, the synergistic effect of the desired bacteria and the nanoparticles used in this formulation increases resistance to biotic and abiotic factors and improves plant growth.

## Supplemental Materials

Supplementary data for this paper are available on-line only at http://jmb.or.kr.

## Figures and Tables

**Table 1:** Treatment during the greenhouse experiments.

Treatments
*R. solani*+ *B. velezensis* nanocomposite beads
*R. solani*+ Free *B. velezensis*
*B. velezensis* nanocomposite beads
Free *B. velezensis*
Nanocomposite beads without bacteria
*R. solani*
Control

**Table 2:** Efficacy of *B. velezensis* VRU1 with free cell and nanoformulation on control of *R.solani* on the bean.

Treatments	Disease Control %
*B. velezensis* nanocomposite beads+ *R. solani*	96.33 ± 1.453^b^
*B. velezensis* bacterium +*R. solani*	80.67 ± 1.202^c^
Nanocomposite beads without bacteria + *R. solani*	25 ± 1.155^d^
*R. solani*	0^e^
Control	100^b^

Mean ± standard errors. Significant differences are according to student's *t*-test with *p* ≤ 0.05.

**Table 3:** Efficacy of *B. velezensis* VRU1 with free cell and nanoformulation on growth parameters in beans plants.

Treatments	SFW (g)	SDW (g)	RFW (g)	RDW (g)
*B. velezensis* nanocomposite beads	3.82^a^	1.84^a^	2.35^a^	1.03^a^
*B. velezensis* nanocomposite beads+ *R. solani*	3.41^b^	1.41^b^	2.25^b^	0.84^b^
*B. velezensis* bacterium	3.11^c^	1.28^c^	2.03^c^	0.53^c^
*B. velezensis* bacterium +*R. solani*	3.08^c^	1.10^d^	1.93^d^	0.42^d^
Nanocomposite beads without bacteria	2.4^d^	0.48^e^	1.52^e^	0.28^e^
Control	2.34^d^	0.4^f^	1.48^e^	0.25^e^
*R. solani*	2.14^e^	0.38^f^	1.32^f^	0.17^f^

SFW: Shoot fresh weight; SDW: Shoot dry weight; RFW: Root fresh weight; RDW: Root dry weight Significant differences are according to student's *t*-test with *p* ≤ 0.05.
