# Supplementary material for: Evaluation of Bacillus velezensis for Biological Control of Rhizoctonia solani in Bean by Alginate/Gelatin Encapsulation Supplemented with Nanoparticles
Source: J Microbiol Biotechnol. 2021 Aug 12;31(10):1373–82. doi: 10.4014/jmb.2105.05001 (PMC9705934; doi:10.4014/jmb.2105.05001)
Supplement: Supplementary file 1 [file jmb-31-10-1373-supple.pdf]

J. Microbiol. Biotechnol.
https://doi.org/10.4014/jmb.2105.05001

Table ANOVA results of Moisture content (H),

Encapsulation efficiency (P), Swelling percentage (I)

Source of variations	df	Mean squares		
		H	I	P
d	5	127.75**	536.54**	1.06ns
Error	12	0.08	54.29	1.89
Total	17			

** -significant ($P < 0.01$), ns- not significant.
